# Retrospective case note review of chronic spontaneous urticaria outcomes and adverse effects in patients treated with omalizumab or ciclosporin in UK secondary care

**DOI:** 10.1186/s13223-015-0088-7

**Published:** 2015-07-21

**Authors:** Sinisa Savic, Alexander Marsland, David McKay, Michael R Ardern-Jones, Tabi Leslie, Olivier Somenzi, Laura Baldock, Clive Grattan

**Affiliations:** St James’s University Hospital, Leeds, LS9 7TF UK; Salford Royal NHS Foundation Trust, Manchester Academic Health Science Centre, University of Manchester, Manchester, M6 8HD UK; Royal Infirmary of Edinburgh, Edinburgh, EH16 4SA UK; University Hospital Southampton, Southampton, SO16 6YD UK; Royal Free London NHS Foundation Trust, London, NW3 2QG UK; St John’s Institute of Dermatology, London, SE1 7EH UK; Novartis Pharmaceuticals UK Ltd, Camberley, Surrey, GU16 7SR UK; pH Associates Ltd, Marlow, SL7 1PG UK; Dermatology Centre, Norfolk and Norwich University Hospital, Norwich, NR4 7UY UK

**Keywords:** Chronic spontaneous urticaria, Ciclosporin, Observational, Omalizumab, Retrospective

## Abstract

**Background:**

Omalizumab is approved in the UK as add-on treatment for chronic spontaneous urticaria (CSU) in patients with inadequate response to H_1_-antihistamines. Ciclosporin is an established but unlicensed 3rd line option for CSU. Two parallel retrospective observational studies were conducted to describe outcomes of treatment and adverse events with omalizumab or ciclosporin for CSU treatment.

**Methods:**

Data from UK specialist centres prescribing omalizumab (five centres) or ciclosporin (three centres) in CSU patients were collected from hospital records by clinical staff and pooled for analysis.

**Results:**

Forty-six patients prescribed omalizumab and 72 patients prescribed ciclosporin were included. Twenty-two (48%) omalizumab-treated patients had paired Urticaria Activity Scores (UAS7), showing a 25.4 point improvement during treatment (P < 0.0001). Paired Dermatology Life Quality Index (DLQI) was available in 28 (61%) omalizumab-treated and 17 (24%) ciclosporin-treated patients. At least a 75% improvement in DLQI score was observed in 79% of omalizumab-treated and 41% of ciclosporin-treated patients, and 65% of omalizumab-treated patients had complete resolution of their quality-of-life impairment (DLQI 0–1) versus 21% of ciclosporin-treated patients. Clinician comments reported symptom clearance in 15/36 (42%) omalizumab-treated and 10/60 (17%) ciclosporin-treated patients. Proportions of patients with adverse events were similar but those for omalizumab resembled CSU symptoms, making causality assignment difficult, whereas those for ciclosporin were consistent with its known adverse effect profile.

**Conclusions:**

Validated patient-reported measures of disease severity and quality of life should be used routinely in CSU management. Based on clinician comments and DLQI scores, symptoms and quality of life showed a greater improvement in the omalizumab-treated cohort than in the ciclosporin-treated cohort.

## Background

In chronic spontaneous urticaria (CSU), non-sedating H_1_-antihistamines are the treatment of first choice, aiming for complete symptom resolution [1, 2]. However, treatment with licensed doses relieves symptoms effectively in fewer than 50% of patients [3]. In cases of non-response, recent European guidelines recommend increasing H_1_-antihistamine dose up to four times the licensed dose [1], but approximately one-third of patients remain antihistamine resistant [4]. The recommended treatment options in patients unresponsive to high-dose H_1_-antihistamines are ciclosporin, omalizumab or montelukast [1]. Other strategies adopted in clinical practice include a change of H_1_-antihistamine, using a combination of H_1_- and H_2_-antihistamines, or addition of dapsone or methotrexate [1]. Although supported by clinical guidelines and some clinical studies, all second and third-line options, with the exception of omalizumab, are unlicensed for CSU. Omalizumab is a recombinant humanized monoclonal anti-IgE antibody which binds to the Fc region of IgE. By sequestering free IgE, it may also indirectly downregulate FcRI receptors on mast cells and basophils, reducing histamine release potential and hence CSU symptoms [5], however the specific mode of action of omalizumab in CSU is currently unknown. In 2014 omalizumab (300 mg by subcutaneous injection every 4 weeks) was licensed as add-on therapy for the treatment of CSU in adult and adolescent (12 years and above) patients with inadequate response to H_1_-antihistamine treatment [6], following studies which demonstrated its efficacy and safety in this group of patients [7–9].

The aim of this investigation was to gather UK real world evidence of third-line treatment options for CSU to facilitate clinical treatment decision making. We conducted two parallel multicentre retrospective case note review studies. In the first study we reviewed patients treated with omalizumab for CSU and in the second, parallel study, we reviewed patients treated with ciclosporin for CSU. We report here the results of both studies, describing the characteristics of patients treated, and the dosing patterns, outcomes and adverse events associated with these treatments.

## Methods

Study sites were identified through clinicians with a specialist interest in CSU, treating patients in dermatology or immunology services. The omalizumab study was conducted in five UK specialist tertiary centres (4 Dermatology, 1 Immunology). The ciclosporin study was conducted in 3 of these 5 centres (2 Dermatology, 1 Immunology).

Patients’ clinical records were reviewed retrospectively by the responsible clinicians and ethical approval for both studies was covered by UK regulations for retrospective medical records review [10]. Research governance approval was obtained for study conduct at each participating centre.

For the present investigation, the omalizumab study comprised consecutive eligible patients first prescribed omalizumab for any form of CU (n = 55) between 19/10/09 and 14/02/14 without selective sampling and we present here those with CSU (n = 46). The eligibility period was pre-licence and omalizumab was accessed via individual funding requests (IFR). The ciclosporin study included consecutive adult patients who received ciclosporin for CSU treatment between 8/08/08 and 31/12/12. Patients with co-existing asthma or atopic eczema were excluded to avoid uncertainty over the indication for prescribing ciclosporin.

All clinical staff were trained in the study requirements and a standardised data collection form and data collection guidelines were used to ensure consistency of methodology across all centres for each study.

The dataset for each study was collected by members of the clinical team from patients’ clinical records, including paper case-notes and electronic hospital databases. Patient and disease baseline characteristics [age, sex, symptoms and severity of CSU, weekly Urticaria Activity Score (UAS7), Dermatology Life Quality Index (DLQI) score, presence of angioedema and severity score, weight, symptom onset and diagnosis dates, co-morbidities and previous CSU medications] were collected from the time of omalizumab/ciclosporin initiation.

The UAS7 is a validated patient reported scoring system [1], which assesses intensity of pruritus (0 = none; 1 = mild; 2 = moderate; 3 = intense) and number of wheals (0 = none; 1 = 1–20 wheals; 2 = 20–50 wheals; 3 = 50+ wheals or large confluent areas of wheals) over 24 h. Daily UAS scores (range 0–6 points/day) are summed over 7 days to create the weekly UAS7 (range 0–42), with higher scores reflecting higher disease activity. The DLQI is a patient-reported validated questionnaire [11] which measures the health-related quality of life (QoL) of adults suffering from a skin disease (range 0–30), with higher scores meaning greater QoL impairment.

Dosing patterns of omalizumab/ciclosporin, outcomes (routinely-administered UAS7, DLQI scores, clinician’s assessments of symptom resolution and adverse events) were collected from the date of omalizumab/ciclosporin initiation to the date of data collection, irrespective of the treatment duration. Disease response according to clinician’s assessment documented in the medical record was classified as either ‘clear of symptoms’, ‘improved symptoms’ or ‘non-responder’ (including patients with no change or worsening of CSU); the best response documented during treatment was recorded as the study outcome measure. Adverse events were extracted from the hospital case notes and patient correspondence (primary care notes were not available). The date, severity, seriousness and assessment of causality of each adverse event were recorded using a consistent set of coded response options.

Analyses were conducted with the available data and the number available for each analysis is stated where data were absent from the clinical record; no imputation of missing data was undertaken.

## Results

### Patients

The baseline characteristics of the patients included in the omalizumab and ciclosporin studies are presented in Table 1. In the omalizumab study, of the 46 patients with CSU (mean age 43.3 years; SD 13.1), 36 (78%) were female. Thirty-six (78%) patients had CSU and 10 (22%) had CSU concurrent with chronic inducible urticaria (CIndU; 7 pressure urticaria, 3 dermographism); four also had documented features of urticarial vasculitis (UV). Angioedema was recorded for 38 (83%) patients; 31 (86%) of those with CSU only and 7 (70%) of those with CSU + CIndU (see Table 1). The ciclosporin study included 72 patients with CSU (mean age 40.5 years; SD 12.8), of whom 61 (85%) were female. Among these, 66 (92%) had a diagnosis of CSU only, while 6 (8%) had CSU + CIndU (three dermographism, two delayed pressure urticaria, one symptomatic dermographism/delayed pressure urticaria and aquagenic pruritus). Fifty patients (69%) had a recorded history of angioedema (see Table 1).

**Table 1:** Baseline patient and disease characteristics

	Omalizumab study, N = 46 (unless otherwise stated)	Ciclosporin study, N = 72 (unless otherwise stated)
Sex		
Male	10 (22%)	11 (15%)
Female	36 (78%)	61 (85%)
Age (years)		
<20	0	3 (4%)
20–29	10 (22%)	12 (17%)
30–39	6 (13%)	16 (22%)
40–49	16 (35%)	28 (39%)
50–59	8 (17%)	7 (10%)
60–69	5 (11%)	4 (6%)
70–79	1 (2%)	2 (3%)
Mean (SD)	43.3 (13.1)	40.5 (12.8)
Weight (kg)	n = 21	n = 24
Mean (SD)	85.3 (27.1)	78.8 (16.2)
Diagnosis		
CSU only	36 (78%)^a^	66 (92%)
CSU + CIndU	10 (22%)^a^	6 (8%)
History of angioedema		
All patients	38 (83%)	50 (69%)
CSU only	31 (86%)	45 (68%)
CSU + CIndU	7 (70%)	5 (83%)
Co-morbidities (not mutually exclusive)		
None	13 (28%)	43 (60%)
Allergic condition	9 (20%)	10 (14%)
Hypertension	8 (17%)	6 (8%)
Asthma	7 (15%)	0^c^
Eczema	7 (15%)	0^c^
Thyroid disorder	3 (7%)	2 (3%)
Depression	5 (11%)	4 (6%)
Anxiety	1 (2%)	4 (6%)
Other	17 (37%)^b^	16 (22%)^b^
Time since first symptoms (years)	N = 42	N = 57
<1	0	12 (21%)
1 < 5	15 (36%)	25 (44%)
5 < 10	16 (38%)	10 (18%)
≥10	11 (26%)	10 (18%)
Not recorded	4	15
Median (IQR)	7.2 (3.7–10.0)	3.2 (1.5–7.6)
Time since diagnosis (years)	N = 37	N = 51
<0.5	0	31 (61%)
0.5 < 1	6 (16%)	11 (22%)
1 < 5	17 (46%)	9 (18%)
5 < 10	12 (32%)	0
≥10	2 (5%)	0
Not recorded	9	21
Median (IQR)	3.8 (1.2–7.5) years	3.7 (2.3–9.1) months
Previous 2nd/3rd line CSU medications (not mutually exclusive)		
2nd line		
Montelukast	23 (50%)	19 (16%)
Dapsone	12 (26%)	3 (4%)
H_2_-antihistamine	10 (22%)	8 (11%)
Sulphasalazine	7 (15%)	1 (1%)
Hydroxychloroquine	8 (17%)	1 (1%)
3rd line		
Ciclosporin	33 (72%)	–
Omalizumab	–	0
Methotrexate	17 (37%)	1 (1%)
Azathioprine	15 (33%)	4 (6%)
Mycophenolate mofetil	12 (26%)	2 (3%)
Tacrolimus	2 (4%)	0
Any 3rd line	39 (85%)	7 (10%)
Others		
UVB light therapy	1 (2%)	0
Rituximab	1 (2%)	0
Cyclophosphamide	1 (2%)	0
Colchicine	3 (7%)	0
Antidepressant	12 (26%)	6 (8%)
Corticosteroids in previous 12 months	29 (74%) (n = 39)	18 (29%) (n = 63)
CSU severity and QoL		
UAS7 Score	n = 27	
0	0	–
1–6 (well controlled)	1 (4%)	–
7–15 (mild disease)	2 (7%)	–
16–27 (moderate disease)	10 (37%)	–
28–42 (severe disease)	14 (52%)	–
Mean (SD)	27.5 (10.4)	–
Median (IQR)	29.0 (20.7–36.1)	–
DLQI Score	n = 32	n = 20
0–1 (disease no impact on QoL)	0	0
2–5 (small impact)	3 (9%)	0
6–10 (moderate impact)	1 (3%)	1 (5%)
11–20 (large impact)	9 (28%)	13 (65%)
21–30 (extremely large impact)	19 (59%)	6 (30%)
Mean (SD)	19.5 (7.1)	17.4 (6.6)
Median (IQR)	21.5 (15.0–24.0)	16.5 (12.0–22.0)

Only data that were recorded in the notes are included so the totals for each field are different for most characteristics.

^a^Two patients showed features of urticarial vasculitis (UV) during their recorded medical history.

^b^Other co-morbidities (not specified in the study DCF) in omalizumab study patients were: Diabetes, 3; Chronic fatigue, 2; Obesity, 2; PVD, 2; osteoporosis/osteopenia, 2; COPD, inflammatory bowel disease, adrenal insufficiency, cardiovascular disease, renal transplant, haemophilia, sarcoidosis, inflammatory arthritis (×1 each). Other co-morbidities (not specified in the study DCF) in ciclosporin study patients were: Diabetes, 4; Autoimmune disorder, 3; Cardiovascular disease, 2 and IBS, Ovarian cysts/fibroids, Cancer, COPD, Chronic fatigue syndrome, pancreatitis, mastitis, stroke/TIA, PE, gall stones, sinusitis and facial nerve palsy (×1 each).

^c^Patients with asthma or eczema were excluded from the ciclosporin study.

### Dosing

Twenty-four (52%) omalizumab-treated patients were initiated on 150 mg and 20 (43%) on 300 mg omalizumab. Eight (17%) patients increased the dose from 150 mg to a maximum of 300 mg and 4 (9%) patients reduced the dose from 300 mg. Dosing frequency was 4 weekly in 32 patients, 3 weekly in one patient, 2 weekly in six patients and >4 weekly in six patients (one patient received only one dose). Omalizumab treatment had been discontinued in 20 patients (43%) at the time of data collection; the reasons for withdrawal were not documented.

The median (range) duration of ciclosporin treatment was 4.8 (0.2–67.1) months overall, 4.4 (0.2–67.1) months for complete courses and 28.1 (17.3–38.6) months for ongoing courses at data collection. The mean daily dose of ciclosporin was 174 mg (SD 85.6). The dose in mg/kg could be calculated for 37 patients; 3 mg/kg was the most common starting dose [8/37 patients (22%)] and 4 mg/kg was the most common maximum dose [10/37 patients (27%)]. During ciclosporin therapy, 27 (38%) patients remained on a constant dose, 15 (21%) increased dose, 13 (18%) decreased dose, 7 (10%) increased then decreased dose, 4 (6%) decreased then increased dose and 6 (8%) had other patterns of dose changes. Ciclosporin had been discontinued in 67 (93%) patients at the time of data collection, with 25 (35%) stopping within 3 months of initiation. Reasons for ciclosporin withdrawal were given for 49/67 (73%) patients, indicating lack of benefit in 23/49 patients (46%), intolerance in 13/49 (26%), successful treatment in 11/49 (22%) and other reasons in 3 (6%).

### Treatment response

#### Omalizumab study

Clinician-documented comments on the best response to omalizumab treatment were available for 36 (78%) patients, of whom 15 (42%) were clear of symptoms, 12 (33%) had some improvement and 9 (25%) had not responded.

**Figure 1:**
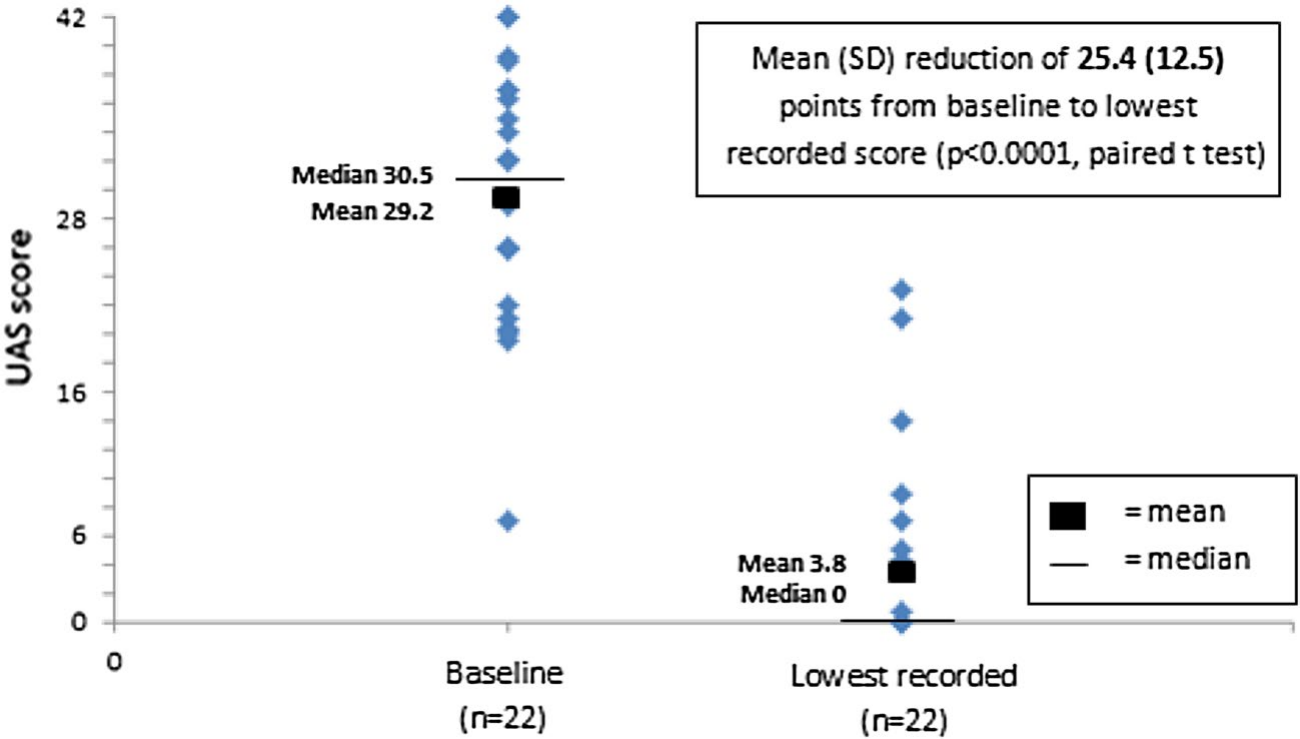
Omalizumab response: within-patient paired UAS7 scores at baseline and lowest recorded on treatment.

**Figure 2:**
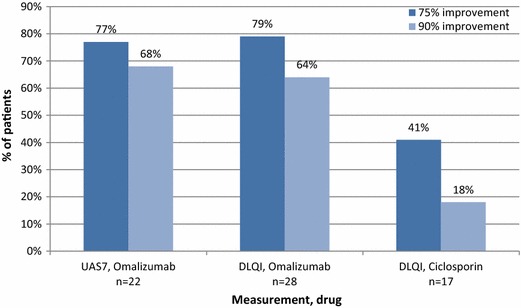
Proportions of patients with 75 and 90% improvement in UAS7 and DLQI from baseline.

The UAS7 score was available for 27 patients at baseline [mean weekly UAS7 27.5 (SD 10.4)]. UAS7 was available for 28 patients at some time following omalizumab initiation [mean UAS7 3.1 (SD 6.3)], and of these 19 (68%) achieved a score of 0 (itch/hives free). The mean weekly UAS7 score for 19 patients with UAS7 recorded at 3 months (±1 month) following omalizumab initiation was 5.8 (SD 9.8). In the 22 patients with paired UAS7 scores, the mean weekly score decreased by 25.4 (SD 12.5) points (P < 0.0001, paired t test) following omalizumab initiation, as shown in Figure 1. The mean of the patients’ percentage improvements was 85%. A reduction in UAS7 score of at least 75% from baseline was observed in 17/22 (77%) patients and a reduction of at least 90% in 15/22 (68%), see Figure 2.

**Figure 3:**
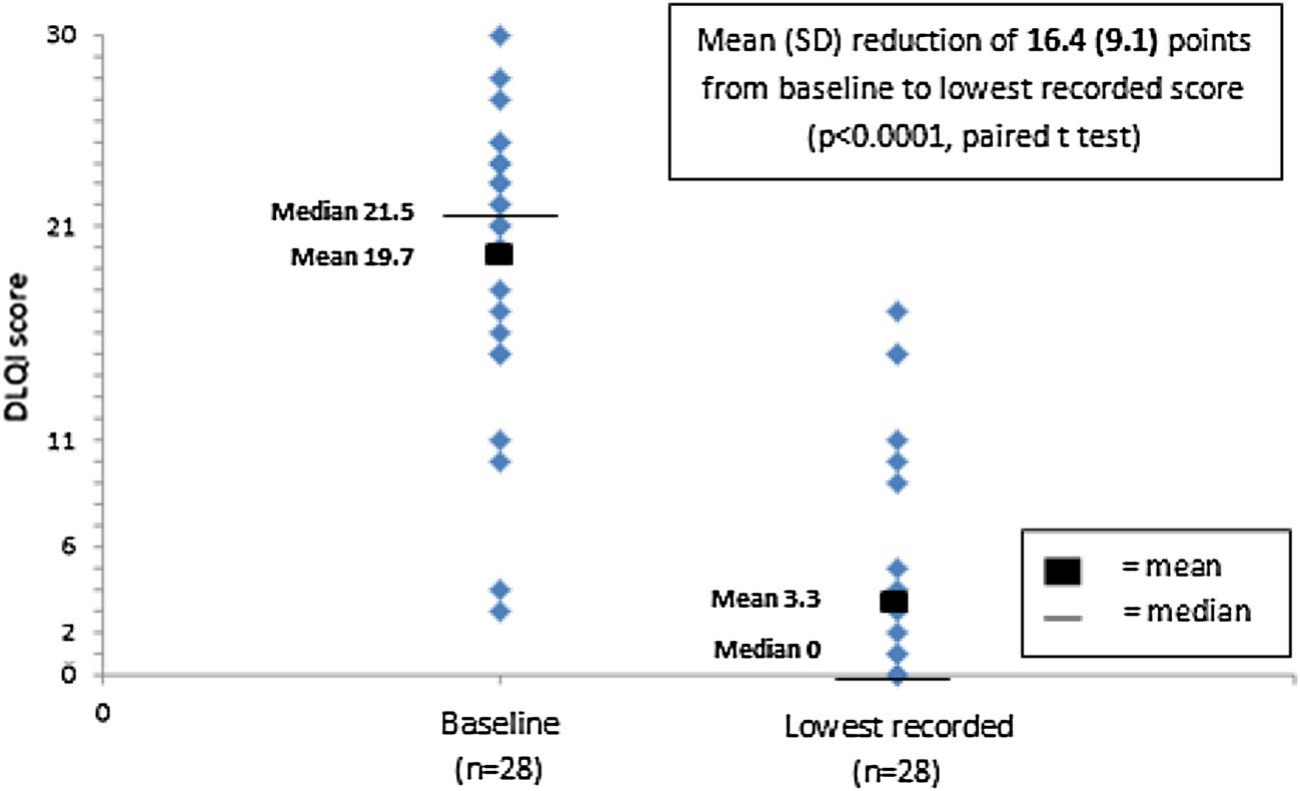
Omalizumab response: within-patient paired DLQI scores at baseline and lowest recorded on treatment.

The impact of CSU on QoL was measured using the DLQI in 32 patients at baseline [mean score 19.5 (SD 5.2)]. DLQI was available in 31 patients at some time during omalizumab treatment [mean score 3.2 (SD 5.2)], and of these 20 (65%) achieved a DLQI score of 0–1 (no impact of disease on QoL). In the 28 patients with paired scores, the mean DLQI score decreased by 16.4 (SD 9.1) points (P < 0.0001, paired t test) following omalizumab initiation, as shown in Figure 3. The mean of the patients’ percentage improvements was 80%. An improvement of at least 75% in DLQI was observed in 22/28 (79%) patients and an improvement of at least 90% in 18/28 (64%; see Figure 2).

#### Ciclosporin study

The best response to ciclosporin according to clinician-documented comments [available for 60 (83%) patients] was clear of symptoms in 10 (17%), improved in 33 (55%) and no response in 17 (28%) patients. The UAS7 score was not used to assess response to ciclosporin.

**Figure 4:**
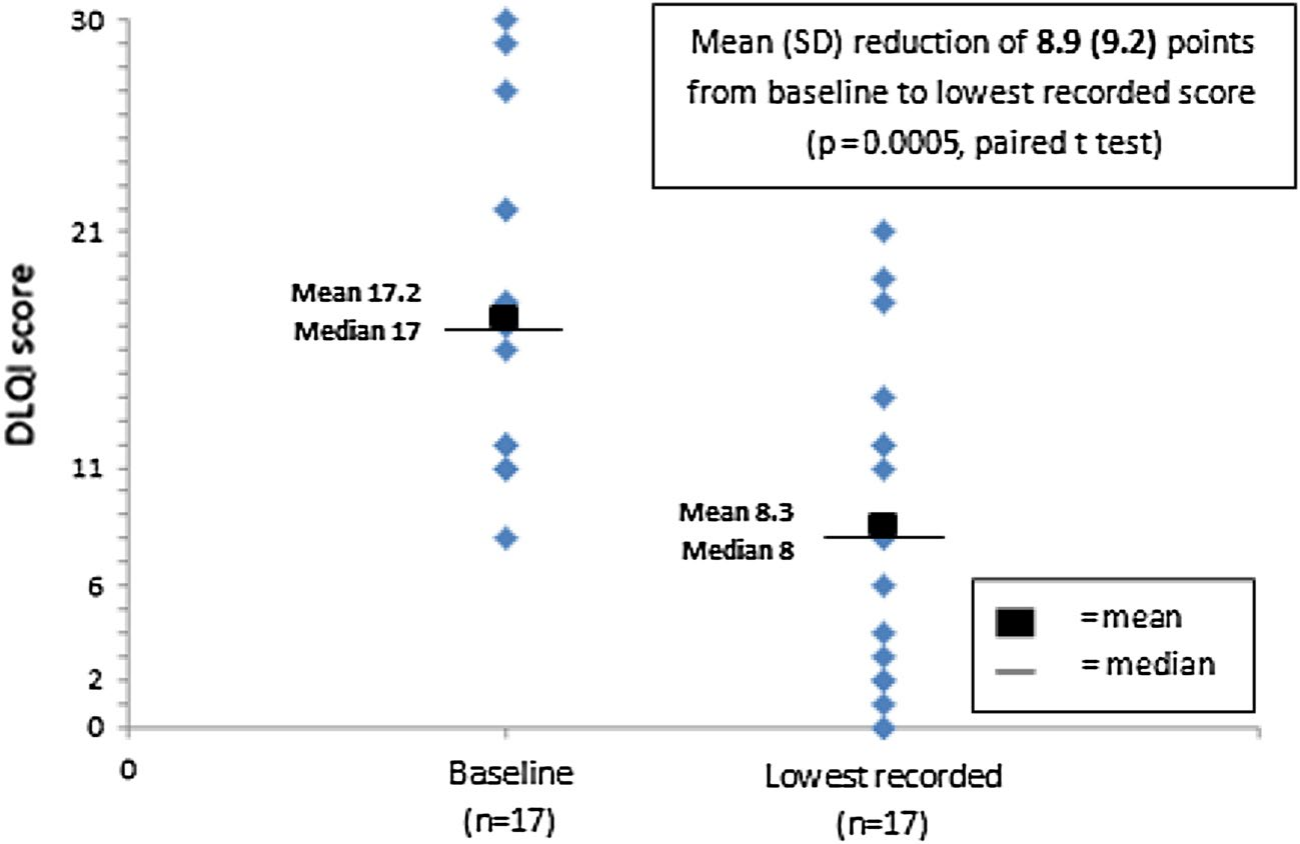
Ciclosporin response: within-patient paired DLQI scores at baseline and lowest recorded on treatment.

DLQI was measured in 20 patients at baseline [mean score 17.4 (SD 6.6)]. DLQI score was available at some time during ciclosporin treatment in 19 patients [mean score 8.5 (SD 7.3)], and of these 4 (21%) achieved a DLQI score of 0–1. In the 17 patients with paired DLQI scores, mean score decreased by 8.9 (SD 9.2) points (P = 0.0005, paired t test), as shown in Figure 4. The mean of the patients’ percentage improvements was 45%. An improvement in DLQI of at least 75% was observed in 7/17 (41%) patients and an improvement of at least 90% in 3/17 (18%; see Figure 2).

### Adverse events

#### Omalizumab study

Potential adverse events were documented during omalizumab treatment for 17 (37%) patients, with a total of 36 events (Table 2). The most common adverse events recorded were skin reactions (eight reports from four patients), angioedema (six reports from three patients), and ‘anaphylaxis’ (three reports from two patients). Overall 35 events were clinician-assessed for severity and causal relationship with omalizumab and judged to be mild in 10 (29%), moderate in 19 (54%) severe in 4 (11%) or not applicable (pregnancy) in 2 (6%). A possible causal relationship with omalizumab was recorded in 20 (57%), with 6 (17%) unrelated and 9 (26%) unknown. Episodes of anaphylaxis were reviewed in detail. One episode of ‘anaphylaxis’ was clinician-rated as ‘moderate’ in severity, and ‘possibly related’ to omalizumab. The episode occurred 2.5 h after omalizumab administration and involved shortness of breath, tongue angioedema and urticaria (blood pressure and pulse were normal, tryptase was not measured), which responded to oxygen, antihistamine, hydrocortisone and self-administered adrenaline in 15 min and the patient was not admitted to hospital. A second episode in the same patient also occurred on the day of omalizumab dosing and was clinician-rated as ‘severe’ and ‘possibly related’ to omalizumab. On this occasion the patient was admitted to hospital overnight; tryptase was slightly raised at 15.5 ng/ml acutely (normal < 11.4 ng/ml) and 13.4 ng/ml the next day. Although both episodes in this patient were documented as ‘possibly related’ to omalizumab, the patient had a history of complex and severe CSU, including recurrent ‘anaphylaxis’ prior to omalizumab, which was incompletely controlled by the therapy. In our opinion the two episodes of ‘anaphylaxis’ in this patient were most likely to have been an exacerbation of the underlying CSU. The third episode of reported anaphylaxis, in a different patient, also occurred on the day of omalizumab injection, and was clinician-rated as ‘severe’. However, we consider this episode not to be consistent with anaphylaxis, although possibly related to omalizumab, based on reviewing the case in detail (symptoms: sharp joint pains and nausea, without severe shortness of breath, hypotension, hives or angioedema; treated by hospital admission for fluids and hydrocortisone). Omalizumab treatment was continued in all three cases.

**Table 2:** Adverse events

AE symptom (not mutually exclusive)	No. symptoms reported	No. patients
Omalizumab		N = 17^a^
Skin (rash, erythema, wheals, eczema, urticaria/itching)	8	4
Angioedema	6	3
Anaphylaxis	3	2
Headaches	2	2
Hot/flushed	2	2
Omalizumab reaction (not specified)	1	1
Palpitations	2	2
Pregnancy	2	2
”Fuzzy head”	1	1
Biliary colic	1	1
Conjunctival haemorrhage	1	1
Erectile dysfunction	1	1
Hair loss	1	1
Injection site reaction	1	1
Sinus bradycardia	1	1
Shortness of breath	1	1
Tachycardia	1	1
Type III Immune complex medicine reaction	1	1
Total—omalizumab	36	–
Ciclosporin		N = 28^a^
Hypertension	8	8
Fatigue/tiredness	6	6
GI	4	4
Headaches	4	4
Altered sensation	3	2
Pregnancy	2	2
Worsening renal function	2	2
Dizziness/collapse	2	2
Excess hair growth	1	1
Increased ESR	1	1
Lower back pain	1	1
Loin pain	1	1
Lumpectomy	1	1
Memory problems	1	1
Miscarriage	1	1
Muscle weakness	1	1
Side effects	1	1
UTI	1	1
Peripheral oedema	1	1
Poor wound healing	1	1
Mild stroke	1	1
Urticarial flare	1	1
Gum soreness and swelling	1	1
Unwell	1	1
Visual disturbance	1	1
Seborrheic keratosis	1	1
Total—ciclosporin	49	–

^a^The number of patients reporting an AE is not the total in this column because some patients experienced more than one adverse event symptom.

#### Ciclosporin study

Potential adverse events were documented during ciclosporin treatment for 28 (39%) patients, with 49 episodes (Table 2). The most commonly reported adverse events were hypertension (eight reports), fatigue/tiredness (six reports), gastrointestinal problems (four reports) and headache (four reports). Of the 49 events, 45 were clinician-assessed for severity, with 24 (53%) rated mild, 16 (36%) moderate, 3 (7%) severe and 2 (4%) not applicable (pregnancy). Thirty (61%) were judged ‘possibly related’ to ciclosporin, 4 (8%) unrelated and 15 (31%) unknown.

## Discussion

These two real world multicentre retrospective observational studies of the treatment of CSU, with either ciclosporin or omalizumab, were carried out in patients for whom early treatment options had failed. The patients were similar in the two studies with respect to age and gender distributions and consistent with the wider population of patients with CSU [12] and patients included in clinical trials of omalizumab [7–9]. The recorded prevalence of unsolicited psychiatric co-morbidities was much lower in both studies than the 30–50% reported elsewhere in patients with CSU [13, 14]. This disparity is possibly due to incomplete recording of psychiatric morbidity and could suggest that this co-morbidity is under-assessed in CSU care in routine practice.

There was considerable variability in initial ciclosporin dosing in the present study and reasons for dose changes were not recorded. Guidelines do not recommend a dose of ciclosporin in CSU but initiating patients on a low dose and up-titrating is an approach that has been advocated to minimise adverse effects while preserving efficacy [15]. Alternatively, studies have used doses of up to 4 mg/kg [16], with 3 mg/kg commonly reported as being effective and tolerable [17] even for long periods [18]. Staged dose reductions from starting doses of 3–4 mg/kg are standard dermatology clinical practice in response to adverse effects. The approach to ciclosporin dosing in the present study (either starting high or starting low) varied between centres rather than between individual patients and seemed to reflect clinician preference.

Due to the absence of any disease severity scores for ciclosporin-treated patients the baseline severity of CSU in the two studies cannot be directly compared, although DLQI, which has been shown to correlate well with UAS7 in patients with CSU [19] was similar at baseline for patients in the two studies. The omalizumab-treated patients in the present study appeared to be characteristic of a more severe disease group as concurrent CSU and CIndU was more prevalent, angioedema more frequent, and more patients had co-morbidities compared with the ciclosporin-treated patients. However, we acknowledge that this may reflect more thorough documentation of medical history in order to obtain omalizumab funding. Omalizumab was also initiated later after initial CSU diagnosis than ciclosporin and most omalizumab-treated patients had tried, and were refractory to, third-line immunosuppressants (Table 1), reflecting the unlicensed status and restricted funding of omalizumab for CSU in the studied period. Thus it is likely that a more severe group was measured in the omalizumab study and as a consequence, direct comparisons of outcomes between the studies of omalizumab and ciclosporin are not possible.

Although formal direct comparison of treatment outcomes is not possible, we can infer some aspects of effectiveness based on the similar baseline DLQI in both groups. The observed improvement in mean DLQI appeared to be greater in patients treated with omalizumab compared with ciclosporin-treated patients, with considerably more omalizumab-treated patients achieving a 75% reduction in DLQI compared with ciclosporin-treated patients (79 and 41%, respectively). The difference was even greater (65 and 18% respectively) in the proportions achieving a 90% reduction in DLQI.

Documented clinicians’ comments were a more widely-available assessment of response to treatment. When reported responses were evaluated categorised as ‘clear of symptoms’, ‘improved symptoms’ or ‘no response’, the data suggested that the response was more often complete, meeting the recommended goal of CSU treatment [1] with omalizumab treatment (42% ‘clear of symptoms’) than with ciclosporin treatment (17% ‘clear of symptoms’). However, caution needs to be exerted in interpretation of these comments due to the subjective nature of the data, and we advocate use of systematic, validated measures of disease severity for all patients with CSU to help clinical teams to monitor optimal disease control by providing consistent outcomes for each treatment prescribed.

Further evidence of the effectiveness of omalizumab in this severely affected cohort was available from patient-reported changes in disease severity (UAS7), with over three quarters (77%) of evaluable omalizumab-treated patients achieving a 75% reduction in UAS7 score and 68% achieving a UAS7 score of 0 (itch and hives free). As this retrospective study was dependent on the routine schedule of clinic visits and clinical practice with respect to formal disease assessment, the data do not allow analysis of the speed of response to omalizumab as the proportion of patients with UAS7 scores even at 3 months was small. However, data from Phase III trials [7–9] and observational studies [20, 21] indicate that responses are rapid, being seen in 53–57% of cases 1 week after the first injection of omalizumab. Although UAS7 was not used to monitor efficacy of ciclosporin treatment, it is notable that 46% of 50 reasons given for stopping ciclosporin in 49 patients, were related to lack of efficacy.

Tolerability appears similar for both drugs, as the overall proportions of patients with documented adverse events were similar (37 and 39% for omalizumab and ciclosporin respectively). There was a broader range of adverse events associated with ciclosporin than with omalizumab and the ciclosporin adverse events reflected its known safety profile. It is notable that many adverse events recorded during omalizumab treatment closely resemble the symptoms of CSU itself, exemplified by the patient with two reported episodes of ‘anaphylaxis’ following omalizumab dosing, which were difficult to separate from the prior history of ‘anaphylaxis’ which was a feature of the CSU in that individual. This observation highlights the difficulty in differentiating natural fluctuations in disease symptoms from adverse events when retrospectively interpreting medical records originally made for clinical care rather than for medical research. As these were small retrospective observational studies and formal recording of adverse events was not of the same standard as required by prospective trials, these data need to be interpreted with caution in association with the broader evidence of tolerability and safety of both drugs.

## Conclusions

It is clear from the present real world data that both omalizumab and ciclosporin treatment were associated with a significant improvement in QoL in patients with CSU. Recently updated European guidelines recommend both ciclosporin and omalizumab as 3rd line CSU treatment options [1]. However, data from these two small real world studies indicate a greater proportion of omalizumab-treated patients achieved at least a 75 and 90% improvement in DLQI compared with ciclosporin-treated patients, suggesting omalizumab treatment is more effective in improving patient QoL. The clinician-documented assessment of response to treatment suggested that more omalizumab-treated than ciclosporin-treated patients achieved clearance of symptoms. Omalizumab was associated with a significant reduction in disease activity in line with findings from recently published Phase III studies and case series [7–9, 19, 20]. The results of these two retrospective observational studies were not directly comparable due to differences between the patient cohorts and lack of consistently recorded validated measures of disease. Adoption of a uniform approach to documentation of CSU disease severity at regular time points, using validated tools, would facilitate comparison of different treatment options and provide evidence for the optimum management of patients with CSU. Despite the limitations of the studies reported here, in the absence of larger prospective studies, these real world data will be beneficial for clinicians in making informed decisions between third-line treatment options for CSU until stronger evidence becomes available.

## Abbreviations

AE: adverse event
CIndU: chronic inducible urticaria
CSU: chronic spontaneous urticaria
CU: chronic urticaria: includes chronic spontaneous urticaria (CSU) (previously known as chronic idiopathic or ordinary urticaria), chronic inducible urticaria (CIndU) (e.g. cholinergic urticaria, delayed pressure urticaria, cold urticaria, solar urticaria, physical urticaria), or a mix of both CSU and CIndU (Zuberbier et al. 2014)
DLQI: Dermatology Life Quality Index
IFR: individual funding request
IQR: interquartile range
NHS: National Health Service
QoL: quality of life
SD: standard deviation
UAS7: Urticaria Activity Score (over 7 days), using retrospective 24 h self-assessed wheal counts
